# CCHCR1 Interacts Specifically with the E2 Protein of Human Papillomavirus Type 16 on a Surface Overlapping BRD4 Binding

**DOI:** 10.1371/journal.pone.0092581

**Published:** 2014-03-24

**Authors:** Mandy Muller, Caroline Demeret

**Affiliations:** 1 Unité Génétique Papillomavirus et Cancer Humain, Institut Pasteur, Paris, France; 2 Université Paris Diderot, Sorbonne Paris Cité, Cellule Pasteur, Paris, France; International Centre for Genetic Engineering and Biotechnology, Italy

## Abstract

The Human Papillomavirus E2 proteins are key regulators of the viral life cycle, whose functions are commonly mediated through protein-protein interactions with the host cell proteome. We identified an interaction between E2 and a cellular protein called CCHCR1, which proved highly specific for the HPV16 genotype, the most prevalent in HPV-associated cancers. Further characterization of the interaction revealed that CCHCR1 binds the N-terminal alpha helices of HPV16 E2 N-terminal domain. On this domain, the CCHCR1 binding interface overlaps that of BRD4, a key mediator of E2 transcriptional activity. Consequently a physical competition occurs between the two proteins for the binding to HPV16 E2, and CCHCR1 interferes with BRD4-mediated enhancement of E2-dependent transcription. In addition, we showed that the interaction with CCHCR1 induced a massive redistribution of HPV16 E2, from a predominantly nuclear to a cytoplasmic localization in dot-like structures, where E2 perfectly co-localizes with CCHCR1. Such a cytoplasmic docking likely interferes with the nuclear functions of HPV16 E2. Upon co-expression of BRD4 and CCHCR1, E2 accumulates both in the nucleus and in the cytoplasm, indicating that for HPV16, both sub-cellular localization and transcriptional functions of E2 may depend on the proportion of both factors within the cell. We provided evidence of a strong induction of the keratinocyte differentiation marker K10 by HPV16 E2, and showed that this activation is compromised by the interaction with CCHCR1. The specific interaction described here could thus impact on the pathogenesis of HPV16. We propose that it could underlie some specific traits of HPV16 infection, such as an enhanced propensity to give rise to lesions evolving toward cancer.

## Introduction

Human papillomaviruses (HPV) have been identified as the causative agents of epithelial lesions, particularly cervical carcinoma, the second leading cause of cancer-related death in women worldwide [1]. HPVs are small double-stranded DNA viruses that infect keratinized epithelia of the skin or mucosa, and are characterized either as low-risk or high-risk according to their association with benign or malignant hyperproliferative lesions respectively. HPV16 is the most prevalent high-risk HPV, accounting for over 50% of HPV-associated cancers.

The HPV life cycle is tightly linked to the differentiation program of the host keratinocytes (reviewed in [2]). The infection first requires that the viral particles reach the dividing cells of the basal epithelial layer, where the viral episomes are maintained at low copy number. The proliferating basal cells then detach from the underlying extracellular matrix, which normally serves as a signal to exit the cell cycle and enter the differentiation process. However, at this point the early proteins interfere with cell cycle exit, leading to a continued proliferation of infected keratinocytes in supra-basal layers and to the induction of dysplasia characteristics of HPV productive lesions. Later, as infected cells move upward in the epithelium, they are eventually committed to differentiation, which is critical for viral genome amplification and synthesis of capsid proteins. The viral particles assemble in the upper layers of the epithelium and are ultimately released by shedding of the uppermost cornified layer.

HPVs are widespread, but in most cases, infections clear spontaneously. However, in a minority of cases, the infection persists and this represents a major risk factor for malignant conversion when associated with high-risk HPV. Therefore, there is a critical need to identify predictive biomarkers to distinguish HPV-infected women at significant risk to develop high-grade lesions and who would greatly benefit from early treatment.

While two prophylactic HPV vaccines and screening programs are available, there is currently no antiviral drug to treat HPV infections and associated diseases. The regulatory E2 protein is considered as a valid candidate target for antiviral compounds [3] since it is a multifunctional early protein with pivotal roles both for the productive life cycle and viral persistence. E2 is composed of two conserved modular domains, the TransActivation Domain (TAD) at the N-terminal end and the DNA-binding Domain (DBD) at the C-terminal end, separated by a non-conserved Hinge region. The E2 protein specifically binds to sites in the viral regulatory region (E2 binding sites, E2BS) and recruits host cell factors necessary to promote replication, regulate transcription and govern proper mitotic segregation of the viral genome. Also, through its association with a number of additional cellular factors, the E2 protein provides cell conditions conductive for the implementation of HPV life cycle along the differentiating epithelium [4].

We have isolated by yeast two-hybrid a protein interacting with HPV16 E2 called CCHCR1 (Coiled-Coil alpha HeliCal Rod protein 1). The CCHCR1 gene (also known as HCR, Pg8, SBP or C6orf18) is highly polymorphic and has been shown to be located within the major psoriasis susceptibility locus [5]. The CCHCR1 protein has been suggested to have a role in the pathogenesis of psoriasis potentially by interfering with keratinocyte differentiation [6]. Notably, CCHCR1 has been associated with the complex regulation of basal keratinocytes proliferation, either as a negative regulator in mouse models [7] or rather as an activator in the context of cancerous cell lines [8]. CCHCR1 therefore principally emerges as a factor influencing the balance between proliferation and differentiation of keratinized epithelia.

In this study, we further characterize the interaction between CCHCR1 and HPV16 E2. We show that E2 binding to CCHCR1 is specific of the HPV16 genotype, the most prevalent HPV in cervical cancer. The interaction is mediated by the alpha-helices of HPV16 E2 N-terminal domain, and the interaction interface of CCHCR1 on E2 overlaps that of BRD4, a key mediator of E2 transcriptional activity. We demonstrate that a competition occurs between CCHCR1 and BRD4 for the binding to HPV16 E2. Consequently, CCHCR1 interferes with BRD4-mediated enhancement of E2 transcriptional activation. The interaction with CCHCR1 induces the docking of HPV16 E2 in the cytoplasm, in dot-like structures typical of the CCHCR1 distribution pattern, thereby possibly opposing the nuclear roles of E2. Upon co-expression of BRD4 and CCHCR1, HPV16 E2 accumulates both in the nucleus where the interaction with BRD4 takes place and in the cytoplasm, indicating that the subcellular distribution of E2 depends on the proportion of these two factors in the cell. We next provide evidence that HPV16 E2 induces a strong increase in the expression of the early differentiation marker cytokeratin 10 in HaCaT cells, and this activation is inhibited by coexpression of CCHCR1. These results suggest that CCHCR1 interferes with at least some aspects of the regulation of keratinocyte differentiation by HPV16 E2.

The highly specific trait of CCHCR1 interaction toward the E2 protein from HPV16 suggests a potential involvement in particular features of HPV16 pathogenesis, possibly related to its high prevalence in HPV-associated cancers. The consequences of such interaction could serve as prognostic biomarkers.

## Materials and Methods

### Expression Plasmids, Adenoviruses

The CCHCR1 ORF was obtained by PCR amplification of CCHCR1 cDNA clones extracted from the Yeast two-hybrid screen, originating from a HaCaT cDNA library (Clontech). Plasmids encoding BRD4 were kindly provided by Cheng-Ming Chiang [9]. All ORFs were cloned into Gateway entry vectors pDON and then transferred into various Gateway compatible destination vectors (pCherry for fluorescence assay, pCiNeo-3XF to generate Flag tagged fusion proteins, pSPICA-N1 for interaction assay, or pCI Neo to express untagged proteins). The plasmids expressing the E2 proteins fused to the different tags used in this study (SPICA-N2- for GPCA interaction assay; 3XFLAG for binding competition assay, GFP for fluorescence) were also constructed using the Gateway recombinational cloning system (Invitrogen) and have been described previously in [10]. The HPV16 E2 proteins with point mutations (N181T, R27D, R37A, I73A and E39A) or deleted of the N-terminal helices were obtained by PCR-directed mutagenesis. Ad-GFPE2 and Ad-GFP constructs were described elsewhere [11].

### Cell Culture and Transfection

293 T and HaCaT (CLS, Cell Lines Service, Germany) cells were routinely maintained in DMEM supplemented with 10% fetal bovine serum at 37°C in a 5% CO_2_ incubator. Where specified, HaCaT cells were grown in Ca^2+^-depleted DMEM. Cells were transfected 24 h after plating by linear PEI (polyethylenimine, Polysciences Inc).

### GPCA

293 T cells were seeded at 35,000 cells per well in 96-well plates. After 24 h, cells were transfected with the following plasmids: pSPICA-N2-E2 encoding for the E2 proteins fused to a Gaussia luciferase fragment (E2-GL2), pSPICA-N1-cellular partner encoding for the cellular proteins fused to the complementary Gaussia fragment (Cellular protein-GL1) (100 ng each), and 10 ng of a CMV-firefly luciferase reporter plasmid to normalize for transfection efficiency. Cells were lysed 24 h post-transfection in 40 μL of Renilla luciferase lysis buffer (Promega) for 30 minutes. The *Gaussia princeps* luciferase activity was measured on 30 μL of total cell lysate by a luminometer Berthold Centro XS LB960 after injection of 100 μL of the Renilla luciferase substrate (Promega). Firefly luciferase was measured on the remaining 10 μL lysate with Firefly luciferase substrate. Gaussia Luciferase activity was reported to Firefly luciferase activity for each sample, giving a normalized Gaussia luminescence. Each normalized Gaussia luciferase activity was calculated from the mean of triplicate samples. For a given pair of proteins (A and B), the normalized Gaussia luminescence of cells coexpressing GL1-A+GL2-B proteins was divided by the sum of normalized Gaussia luminescence of each partner coexpressed with the complementary empty plasmid reflecting the interaction intensities as Normalized Luminescence Ratio (NLR) as follows: GL1-A+GL2-B/(GL1-A+GL2)+(GL1+GL2-B). Competition assays were performed by plating 2.5×10^5^ 293 T cells in12-well plates, followed 24 h later by transfection of 0.5 μg of expression plasmids for each partner (partner GL1-A and GL2-B of the interaction evaluated in GPCA, and a 3XFlag-tagged challenging protein). 40 h post transfection, cells were harvested and subjected to Renilla luciferase assay according to the manufacturer's instructions (Promega).

### Co-immunoprecipitation and Western blot

293 T cells were grown in 6-wells plates and transfected with 1.5 μg of indicated expression plasmid. 30 h post-transfection cell lysates were prepared by incubating cell pellets in lysis buffer (NaCl 150 mM, NP40 0.5%, Tris-HCl pH 8 50 mM, DTT 1 mM, protease inhibitors) for 15 min at 4°C, followed by centrifugation at 10,000 rpm for 15 min at 4°C. For immunoprecipitation, cell lysates were precleared for 2 h with protein A/G-sepharose beads and incubated overnight with Mouse anti-FLAG antibody at 4°C. Immune complexes were collected on protein A/G-agarose beads, washed three times with lysis buffer, eluted with sample buffer and used for western blot analysis. For Western blot, cell lysates were separated on SDS-PAGE acrylamide gels than transferred to nitrocellulose membranes and treated with a primary antibody overnight at 4°C followed by the appropriate HRP-conjugated secondary antibody for 1 h at room temperature. Antibodies used as follows rabbit anti-GFP (1∶5000, Torrey Pines biolabs); rabbit anti-CCHCR1 (1∶1000, Epitomics): Mouse anti-Flag (1∶5000, Sigma); mouse anti-αtubulin (1∶1000, Calbiochem)

### Fluorescence Analysis

HaCaT cells expressing GFP and mCherry-fused proteins were fixed 24 h post-transfection in 4% paraformaldehyde for 30 min at 4°C, permeabilized with 0.1%Triton-containing PBS and stained for 30 min with DAPI. Cells were washed in PBS then mounted with CitiFluor. Fluorescent images were acquired using a ZEISS Apotome microscope.

### Transactivation Assays

5×10^5^ HaCaT cells were plated then transfected 24 h later with 100 ng of the E2-dependent reporter plasmid pTK6E2BS described in [10]. Other expression vectors included 25 ng of a Renilla luciferase-expressing plasmid (POLIIIR) as an internal control for normalization purpose, 100 ng of pCINeo-driven HPV16 E2, and 0.8 μg of 3XFLAG-tagged challenging proteins BRD4 and CCHCR1. Cells were harvested 30 h post-transfection, lysed in Passive lysis buffer according to manufacturer's instructions and the luciferase activities were measured with Dual Glo substrates (Promega).

### Differentiation assay

For the differentiation assay, HaCaT were maintained in calcium free DMEM (Invitrogen) in order to keep HaCaT cells in an undifferentiated state [12], [13]. Infections with recombinant adenoviruses expressing the GFP-E2 fusion proteins or GFP only were done at a multiplicity of infection of 250, in 1 mL of DMEM complemented with 4 μM polybrene for 1 h at 37°C. The medium was then replaced by fresh medium with 10% fetal bovine serum. Cells were collected 24 h or 48 h later and subjected to RNA extraction.

### RT-qPCR

Total RNA was isolated by TRIzol Reagent (Invitrogen) according to the manufacturer's instructions, and used for cDNA synthesis by Superscript II (Invitrogen). The cDNAs were used as templates for quantitative PCR using SYBR Green PCR master mix (Applied Biosystems). Primers used for RT-PCR are listed in Table 1. The ΔΔCt method of was used to calculate the fold changes as described in [13], [14]. Results are represented as relative change over mock-transfected or Ad-GFP infected controls wells.

**Table 1:** Sequences of primers used for qPCR experiments.

K14	5′-GCGGATGACTTCCGCACCAAGTATGAG-3′
	5′-CCTTCAGGCTCTCAATCTGCATCTCC-3′
K10	5′-GATGTGAATGTGGAAATGAATGCTGCCC-3
	5′-GTTCCTTGCTCTTTTCATTGAACCAGGC-3′
TGM1	5′-CAGTGCTGCGCTGCCTGGGTC-3′
	5′-CCGGCCTCTTCATCCAGCAGTC -3′

### Stastistical Analysis

All results are expressed as means ± S.E. of triplicate measurements with all experiments being repeated independently at least three times. Unpaired Student's *t* test was used to evaluate the statistical difference between control and treated groups. Values of *p*<0.05 were considered significant.

## Results

### The HPV16 E2 protein associates with CCHCR1

**Figure 1:**
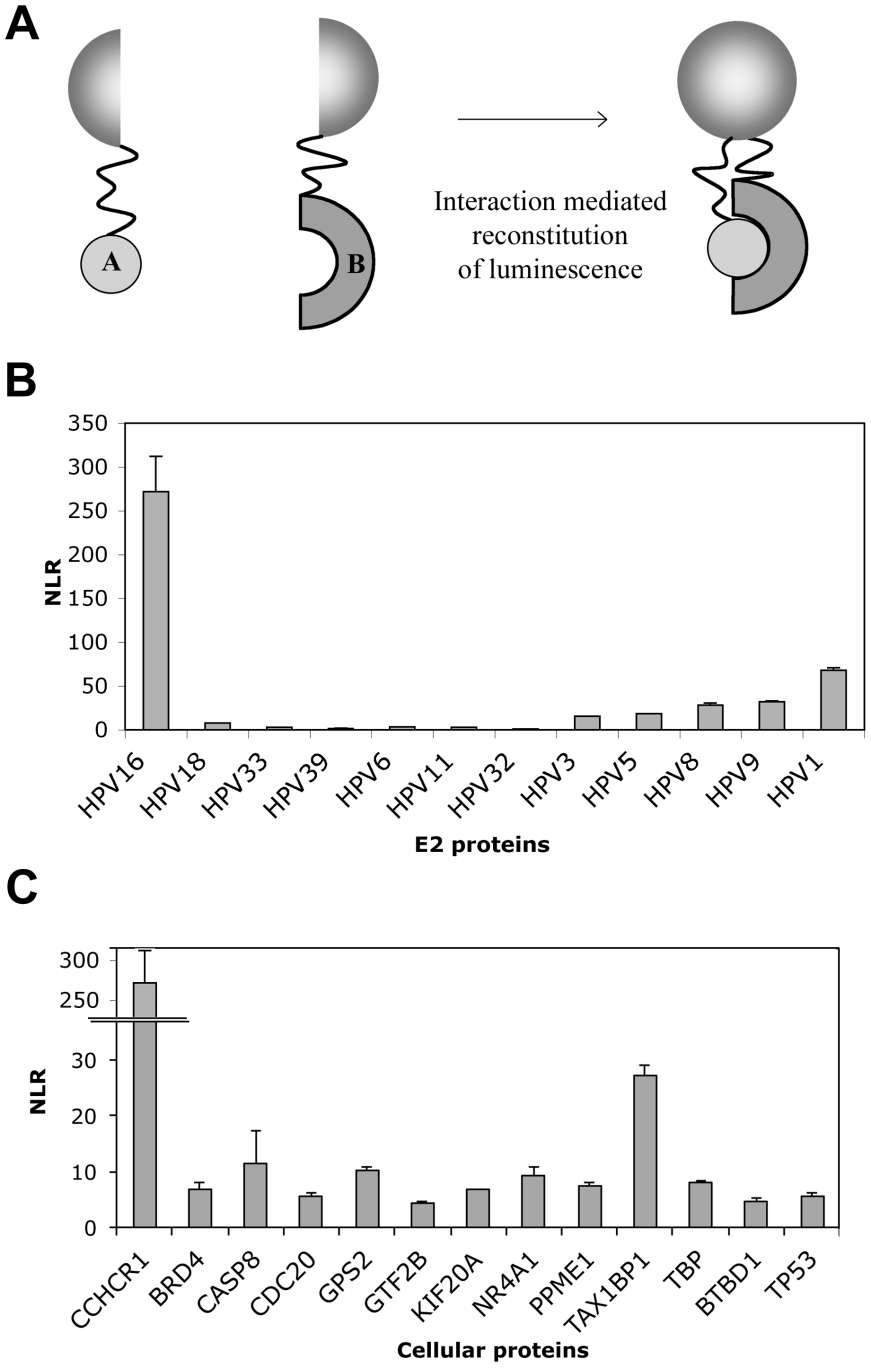
Interaction between HPV16 E2 and CCHCR1. (A) Schematic representation of the GPCA technique. Two proteins A and B are co-expressed in 293 T cells as fusions with two inactive and complementary fragments of the *Gaussia princeps* luciferase. An interaction between A and B proteins reconstitutes the *Gaussia* enzymatic activity by bringing in close proximity both fragments. The interaction level is estimated from a NLR (Normalized Luminescence Ratio) corresponding the *Gaussia* luciferase activity measured when both fusion proteins are expressed divided by the sum of background activities generated by each fusion protein expressed with the empty complementary vector (see [16] for further details). (B) CCHCR1 binding to a panel of E2 proteins. The interactions between 12 E2 proteins and CCHCR1 were measured in GPCA. **, *p*<0.01 versus the interaction between HPV16 E2 and CCHCR1. (C) Interactions between HPV16 E2 and a panel of known HPV16 E2 interacting partners. The interactions between HPV16 E2 and 13 literature-curated known interactors of this E2 protein were assessed in GPCA. **, *p*<0.01 versus the interaction between HPV16 E2 and CCHCR1.

With the goal to decipher the functions of the E2 proteins emerging from their interactions with the host proteome, we recently performed a large-scale identification of E2 partners by yeast two-hybrid with 12 different genotypes of HPVs [10]. In these screenings, one particular cDNA was picked up 13 times with the E2 protein from HPV16. It contained the ORF for a protein called CCHCR1 downstream of the GAL4-TAD-encoding cDNA, but a frameshift between the two ORFs led us to exclude it for further validation in the course of our previous comparative E2 interactomic study [10]. However, given the acknowledged ability of yeast to bypass frameshifts and rectify reading frames, the hit frequency for this clone, and because CCHCR1 has previously been identified as an interaction partner of HPV16 E2 by Olejnik-Schmidt *et al.* [15], we figured it was worth to assess the validity of this interaction. In order to increase the stringency of the validation, we chose to use a *Gaussia Princeps* Fragment Complemention Assay (GPCA), a cell-based protein-protein interaction detection method. This method both demonstrated a high reliability for sensing protein-protein interactions [16], and was previously used to conduct the comparative E2 interactomic study [10], which we thought could be beneficial to interpret the present interaction data. GPCA is based on the reconstitution of a luciferase signal upon interaction between two proteins co-expressed in 293 T cells in fusion with two inactive and complementary fragments of the *Gaussia* luciferase enzyme (Fig. 1A). The interaction intensity is deduced from a NLR (Normalized Luminescence Ratio), which takes into account the background Luciferase signals generated by each fusion protein alone, as well as the transfection efficiency [10], [16]. An NLR value of 3.5 has been determined previously as the cut-off for positive interactions [16]. We therefore transferred the CCHCR1 sequences collected in the clones obtained by yeast two-hybrid into a vector compatible with GPCA. These sequences encoded a unique polypeptide spanning from amino acid 137 to the stop codon of the full-length CCHCR1 protein. This cDNA may represent a natural isoform of CCHCR1 since its expression is highly complex, with a plethora of differently spliced mRNA reported in the Ensembl database, the longest isoform being 782 amino acids long.

To evaluate CCHCR1 binding to different HPV genotypes, we decided to carry out the validation step with the 12 E2 proteins used in the aforementioned comparative E2 interactomic study [10]. Figure 1B represents the NLR detected for each protein pair. It first shows that the interaction between CCHCR1 and HPV16 E2 could be easily detected in GPCA, as shown by a high NLR of about 250 generated upon their co-expression. These results strongly argue that CCHCR1 is a true binding partner of HPV16 E2, given that this interaction was sensed by two orthogonal methods, which highly increases the robustness of interaction data [17]. Except HPV16 E2, none of the mucosal HPV E2 proteins tested (HPV6, 11, 18, 32, 33, 39) were able to interact with CCHCR1. On the other hand, the cutaneous HPV E2 proteins (HPV-1, 3, 5, 8, 9) generated some level of NLR, but it was overall far below that of HPV16 E2 (Fig. 1B). These observations led us to conclude that the interaction between CCHCR1 and E2 has a high degree of specificity toward HPV16. In addition, the interaction of HPV16 E2 with CCHCR1 generated by far the highest NLR when compared by GPCA with a panel of previously identified HPV16 E2 partners selected from the literature [4] (Fig. 1C). In good agreement with the literature, all *a priori* positive interactions scored an NLR above 3.5, indicative of positive interactions [16]. Nevertheless, they appeared globally weak in comparison to the interaction between HPV16 E2 and CCHCR1. We ascertained that the high NLR associated specifically with HPV16E2/CCHCR1 interaction was not due to a higher amount of proteins in comparison to other E2 proteins or cellular factors (Fig. S1).

Altogether, the results obtained by GPCA indicate that the interaction between HPV16 E2 and CCHCR1 is specific. Since it was first identified by yeast two-hybrid, then validated here by a protein-fragment complementation (GPCA), both methods acknowledged to primarily detect binary interactions, we infer that this interaction is likely to be direct. However, *in vitro* interaction assay with purified proteins would be necessary to formally determine this issue.

### CCHCR1 interacts with HPV16 E2 N-terminal alpha-helices and interferes with the binding of BRD4

**Figure 2:**
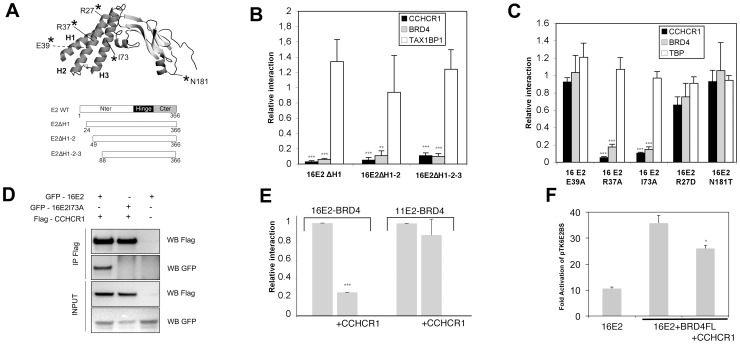
Mapping of the CCHCR1 binding interface on HPV16 E2. (A) top: schematic representation of HPV16 E2 N-terminal domain picturing the position of point mutations used. The E39 amino acid is shown with a dot line since it faces the opposite side of the helices. H1, H2, H3 are respectively alpha-helix 1, 2 and 3. Bottom: diagrams of the HPV16 E2 deletion mutants. (B) Interactions between HPV16 E2 deletion mutants and CCHCR1, BRD4 and TAX1BP1 tested by GPCA. Results are represented relative to the interaction with wild type HPV16 E2. **, *p*<0.01; ***, *p*<0.001 versus the interaction with the wild type HPV16 E2. (C) Interaction between HPV16 E2 point mutants and CCHCR1, BRD4 and TBP tested by GPCA. Results are represented as relative to the interaction with the wild type HPV16 E2 protein. ***, *p*<0.001 versus the interaction with the wild type HPV16 E2. (D) 293 T cells were co-transfected with expression plasmids for Flag-CCHCR1 and GFP-HPV16 E2 WT or I73A as indicated. Cell were lysed and subjected to immunoprecipitation (IP) using anti FLAG antibody followed by western Blotting (WB) with anti FLAG or anti GFP antibodies as indicated. (E) Interaction between HPV16 E2 or HPV11 E2 and BRD4 was assessed in GPCA, in the presence of CCHCR1 as a challenging protein where indicated. Results are reported to NLR values obtained when an empty plasmid was used instead of the challenging protein. ***, *p*<0.001 versus the interaction without CCHCR1 as a challenger. (F) HaCaT cells were transfected with an E2-reponsive luciferase reporter plasmid (pTK6E2BS) in the presence of HPV16 E2, BRD4 plus CCHCR1 where indicated. Fold activation are given relative to promoter activity without E2; *, *p*<0.05 versus experiments without CCHCR1.

When detecting the interaction between CCHCR1 and HPV16 E2 by yeast two-hybrid, Olejnik-Schmidt and colleagues identified the N-terminal domain of E2 as being responsible for the interaction [15]. To further characterize the interaction interface of CCHCR1 on HPV16 E2, we first performed serial deletions of E2 N-terminal alpha helices (schematized in Fig. 2A), and assessed CCHCR1 binding by GPCA. As shown in Figure 2B, as soon as the first helix is deleted, the binding of HPV16 E2 to CCHCR1 is lost. This parallels the interaction with BRD4, which is mediated by the N-terminal helices of E2 [18]. In contrast, the deletion of all three helices does not substantially impact on HPV16 E2 binding to TAX1BP1, thereby confirming the integrity of the deletion mutants.

We next studied the interaction of CCHCR1 with point mutants of HPV16 E2 N-terminal domain to define more precisely the localization of CCHCR1 binding interface. We used E2-R37A and E2-I73A, mutated at amino acids located on one side of the surface formed by the N-terminal helices [19] and known to be pivotal for BRD4 binding [18]; as well as E2-E39A where the mutated amino acid is exposed at the opposite helices surface, and is essential for the binding of the E1 viral helicase. The mutation of E39 had no effect on HPV16 E2 binding to CCHCR1 (Fig. 2C). In contrast, mutations R37A and I73A drastically inhibited the interaction with CCHCR1, as well as with BRD4 as expected (Fig. 2C). TBP, used here as a control since it interacts with the C-terminal domain of E2, consistently bound all point mutants of HPV16 E2 (Fig. 2C). In addition, the mutated proteins accumulated to similar levels as the wild-type HPV16 E2, as assessed by western blot analysis (Fig. S2). These results support the conclusion that the interaction with CCHCR1 involves a surface of the N-terminal alpha helices on HPV16 E2 overlapping the binding interface of BRD4. The co-crystal of HPV16 E2 in complex with the carboxy-terminal domain of BRD4 (BRD4 CTD) revealed that the interaction spans over the three N-terminal alpha-helices of E2 [18]. One can hypothesize that the interaction between CCHCR1 and E2 would similarly cover a large part of the helices surface. Keeping in mind that this interaction is specific for HPV16, we mutated the amino acid R27 since it is found only in HPV16 E2 and is exposed on the same side of the helices than R37 and I73 (Fig. 2A). However, this mutation had only little effect on the interaction between HPV16 E2 and CCHCR1. Also, we noticed that HPV16 E2 exhibited an asparagine at position 181 whereas a threonine was conserved among all other E2 proteins at the equivalent position. We reasoned that the binding interface of CCHCR1 could extend over a large area of HPV16 E2 N-terminal domain, and we therefore tested whether this amino acid could contribute to the interaction despite being localized outside the helical part. Mutating this asparagine to threonine did not affect the binding of CCHCR1 on HPV16 E2 (Fig. 2C) and therefore amino acids R27 and N181 are unlikely to participate to the specific nature of the interaction between CCHCR1 and HPV16 E2.

To confirm by another method the interaction between HPV16 E2 and CCHCR1, we carried out co-immunoprecipitation assays. We showed that CCHCR1 specifically co-precipitated the wild-type HPV16E2 protein, but not 16E2I73A point mutant, corroborating the above results (Fig. 2D).

Since BRD4 and CCHCR1 share a common binding interface on HPV16 E2, we next wanted to determine if they could compete with each other. To do so, we assessed by GPCA the interaction between HPV16 E2 and BRD4 in the presence of CCHCR1. As shown in Figure 2E, the addition of CCHCR1 induces a 5-fold decrease of the NLR, indicating a reduced interaction between HPV16 E2 and BRD4 in presence of CCHCR1. When the same experiment was performed with HPV11 E2, known to interact with BRD4 but not with CCHCR1, the addition of CCHCR1 had only little effect on its association with BRD4. These results demonstrate that CCHCR1 direct interaction with HPV16 E2 interferes with the binding of BRD4. Taken together, our data indicate that BRD4 and CCHCR1 competitively bind to HPV16 E2, in line with their overlapping binding interface over the N-terminal helices.

The interaction with BRD4 is necessary for E2-dependent transcription [20]. We therefore analyzed the functional impact of CCHCR1 competition with BRD4 using a synthetic luciferase reporter containing E2 binding sites upstream of a minimal TK promoter (pTK6E2BS). We could show, in agreement with previous reports, that expression of BRD4 enhanced E2-mediated transcriptional activation, and that upon co-expression of CCHCR1, the activation of E2-dependent transcription by BRD4 was reduced (Fig. 2F). In contrast, CCHCR1 did not interfere with the enhancement of HPV18 E2 transcriptional activity by BRD4 (Fig. S3). These results show that CCHCR1 binding to HPV16 E2 interferes with the positive role of BRD4 on E2 transcriptional properties, thereby providing an additional assessment of the competitive binding of CCHCR1 and BRD4 to HPV16 E2.

### The interaction between CCHCR1 and HPV16 E2 impacts on the regulation of keratinocytes differentiation

The role of CCHCR1 in keratinocytes has been linked to the regulation of their proliferation status. It has been previously stated by Tiala *et al.* that, in transgenic mice, CCHCR1 is a negative regulator of keratinocyte proliferation [7], but in cell lines, CCHCR1 expression has been found to correlate with the presence of the proliferation marker Ki-67, and is therefore rather pro-proliferative [8]. These studies suggest that CCHCR1 may have a different influence on the regulation of proliferation according to the cell context.

**Figure 3:**
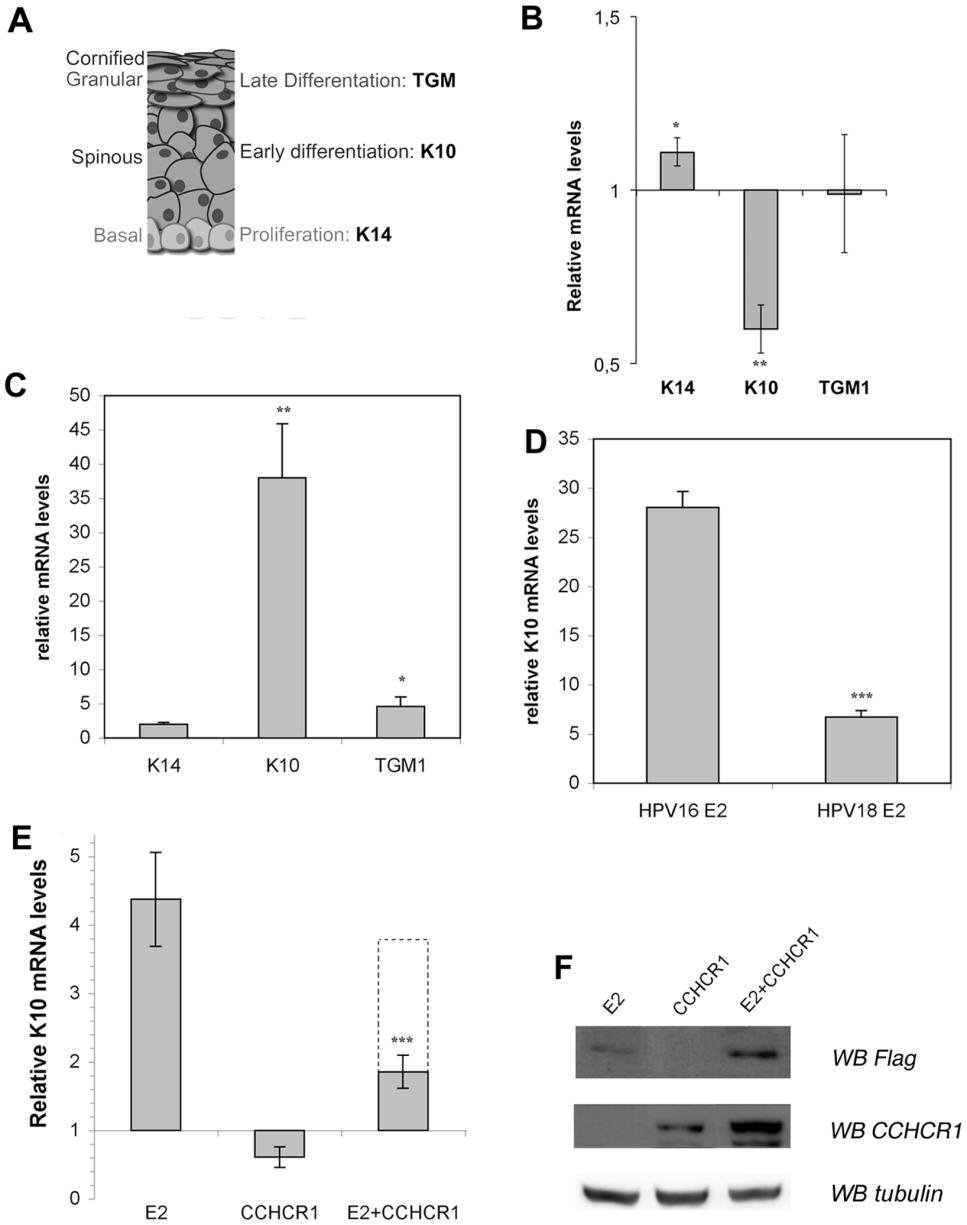
CCHCR1 inhibits the activation of early differentiation by HPV16 E2. (A) Schematic representation of the differentiation of a keratinized epithelium, with studied markers outlined. (B) HaCaT cells were transfected by a CCHCR1 expression plasmid, and the mRNA levels of three differentiation markers were subsequently monitored by qRT-PCR. Results are represented as relative to mock- transfected cells (empty plasmid). *, *p*<0.05; **, *p*<0.01 versus mock-transfected cell experiments. (C) HaCaT cells were infected by Ad-GFP16E2 and the mRNA levels of three differentiation markers were subsequently monitored by qRT-PCR. Results are represented as relative to cells infected with Ad-GFP. *, *p*<0.05; **, *p*<0.01 versus cells infected with Ad-GFP. (D) HaCaT cells were infected by Ad-GFP16E2 or Ad-GFP18E2 and mRNA levels of K10 were subsequently monitored by qRT-PCR. Results are represented as relative to Ad-GFP expressing cells. ***, *p*<0.001 versus cells infected with Ad-GFP16E2. (E) HaCaT cells were transfected by 3XFLAG-HPV16 E2 and CCHCR1 expression plasmids as indicated and the subsequent effect of the mRNA levels of K10 was monitored by qRT-PCR. Results are represented relative to mock-transfected cells. The dotted line represents the K10 mRNA levels expected by simply combining the levels obtained in the presence of each E2 and CCHCR1 protein separately. ***, *p*<0.001 versus cells infected with HPV16 E2 only. (F) HaCaT cells were transfected as in (E) and western blots were performed on total cell lysate with anti FLAG or anti CCHCR1 antibodies, then with anti-tubulin antibody as loading control.

Since the switch between proliferation and differentiation is known to be a key process hijacked by HPVs to promote their replication, we wished to examine how the interaction between CCHCR1 and HPV16 E2 could impact on this process. Previous studies have shown that the HaCaT keratinocytes conserved some aspects of their differentiation capabilities when cultured in low-calcium concentration [12], [13]. Experiments were therefore carried out in this cell line. We first verified that in our culture conditions, addition of calcium correctly mimicked the induction of a differentiation program as stated in earlier publications (not shown). We next wished to understand the effect of CCHCR1 in our experimental settings. For that purpose, CCHCR1 was ectopically expressed in HaCaT cultured in low-calcium concentration. We then monitored by qRT-PCR the mRNA levels of Keratin 14 (K14), which is a marker of basal proliferative keratinocytes, of Keratin 10 (K10) and Transglutaminase 1 (TGM-1), chosen to be hallmarks of keratinocytes differentiation (Fig. 3A). As shown in Figure 3B, expression of CCHCR1 leads to a weak activation of K14, and to a repression of the early differentiation marker K10, indicating that CCHCR1 would rather have a pro-proliferation effect in the HaCaT cell line. In contrast, expression of CCHCR1 had no significant effect on the mRNA levels of the late differentiation marker TGM1. In the reciprocal experiment where CCHCR1 expression was knocked-down using a siRNA, we could observe a decreased K14 and an enhanced K10 expression (data not shown). These results confirm that CCHCR1 affects the expression of proliferation and early differentiation markers, and is likely to regulate the balance between proliferation and differentiation in a pro-proliferative direction.

We next wished to examine the effect of HPV16 E2 on keratinocytes differentiation in our experimental settings. HaCaT cells were infected by recombinant adenoviruses expressing GFP-16E2 or GFP only and were assessed for expression of differentiation markers. As shown in Figure 3C, HPV16 E2 drastically enhanced the expression of K10 (more than 35 times). In the same conditions, HPV16 E2 had no or a weak effect on the expression of Keratin 14 (K14) and (TGM1), indicating that it primarily induces early differentiation. The E2 protein from HPV18 expressed from recombinant adenoviruses had only a modest effect on K10 expression, when compared to HPV16 E2 (Fig. 3D). The strong activation of K10 therefore appears to be characteristic of the E2 protein from HPV16, so we figured that it might be related to its specific interaction with CCHCR1. We conducted co-transfection experiments to evaluate the consequences of the interaction between HPV16 E2 and CCHCR1 on K10 expression (Fig. 3E). Use of a plasmid instead of a recombinant adenovirus to express HPV16 E2 reduced the transfection efficiency, but still induced a 4-fold activation of K10 (Fig. 3E), whereas expression of CCHCR1 alone resulted in the repression of K10 as previously shown in Figure 3B. Upon co-expression of CCHCR1, K10 activation by HPV16 E2 was reduced to a level of 1.85 fold (Fig. 3E). Such decrease is more drastic than what would be expected from the simple combination of CCHCR1 negative and HPV16 E2 positive effects on the expression of K10 (dotted line in Fig. 3E). Importantly, we verified that the decrease of E2-mediated K10 activation was not due to a reduced level of HPV16E2 protein (Fig. 3F). In fact, HPV16 E2 rather better accumulated in the presence of co-expressed CCHCR1, which was observed with any protein (not shown).

These observations indicate that CCHCR1 interferes with HPV16 E2-mediated activation of K10. The mutated 16E2 protein I73A does not have any effect on the expression of the differentiation markers tested, in the presence or not of co-expressed CCHCR1 (Fig. S4), indicating that the effect on K10 expression is transcriptional. Taken together, our results show that HPV16 E2 has a role in promoting early differentiation of infected keratinocytes but CCHCR1 is opposing this effect and would rather promote proliferation.

### CCHCR1 relocalizes HPV16 E2 into the cytoplasm

**Figure 4:**
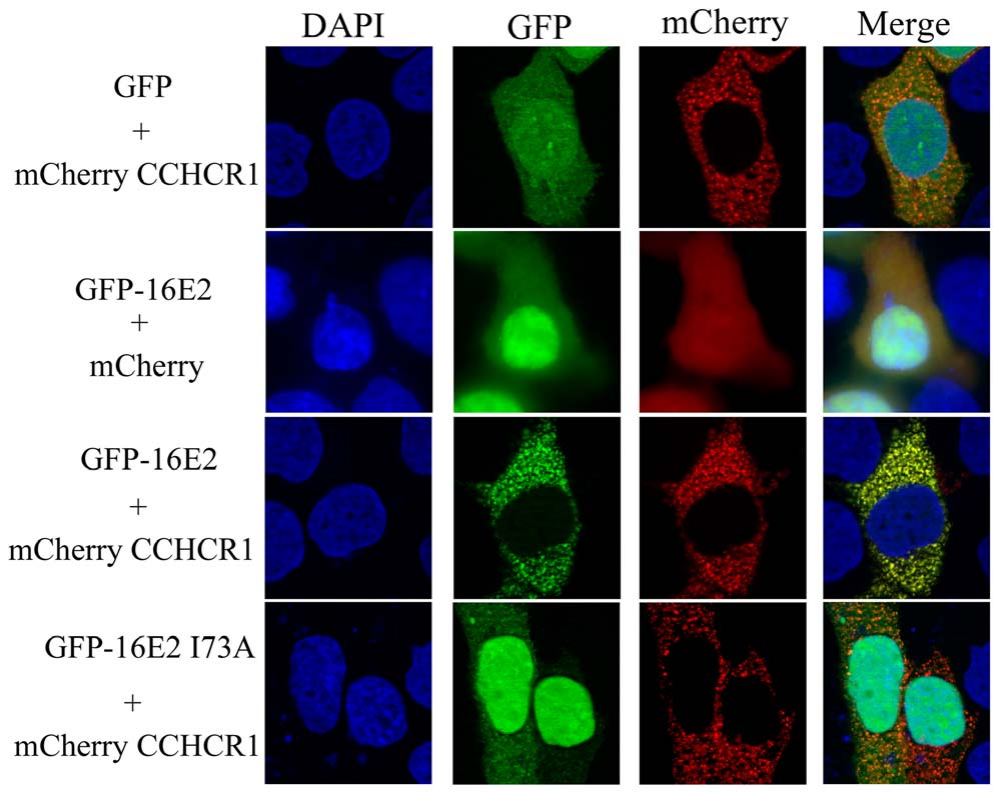
CCHCR1 docks HPV16 E2 into cytoplasmic dot structures. HaCaT cells were co-transfected with the expression plasmids for GFP or the indicated GFP-E2 proteins, and mCherry-CCHCR1 or mCherry alone. After fixation, the cells were subjected to fluorescence microscopy after counterstaining of the nucleus with DAPI. From top to bottom: Ectopic expression of CCHCR1 shows punctate staining (red) in the cytoplasm while HPV16 E2 displays a diffuse pattern both in the nucleus and in the cytoplasm. When co-expressed, the two protein signals overlap in cytoplasmic dots typical of CCHCR1 expression. The non-interacting E2 protein mutated HPV16 E2 I73A showed no delocalization from the nucleus.

**Figure 5:**
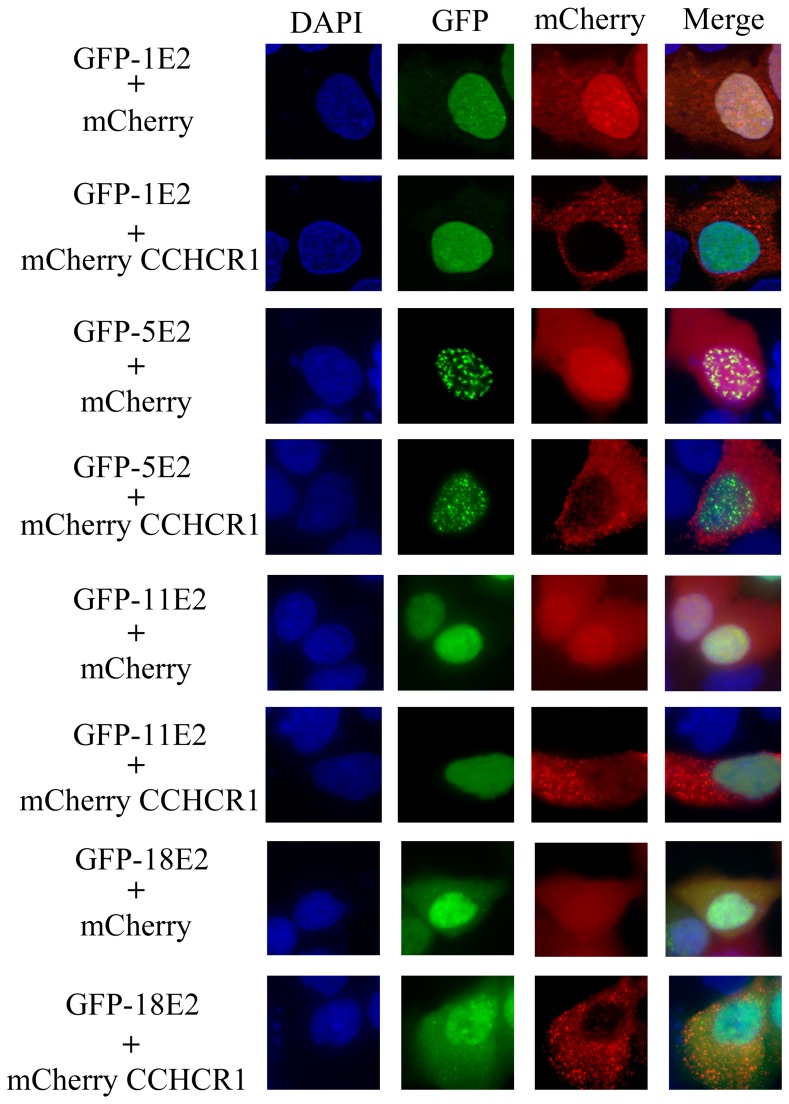
CCHCR1 does not affect the distribution of non-interacting E2 proteins. HaCaT cells were co-transfected with the expression plasmids for GFP or the indicated GFP-E2 proteins, and mCherry-CCHCR1 or mCherry alone and processed as in Figure 4. Four E2 proteins (HPV1, 5, 11 and 18) not able to interact with CCHCR1 were studied, and no change in their subcellular localization could be observed in the presence of CCHCR1.

To get further insights into how CCHCR1 impacts on HPV16 E2 functions, we conducted fluorescent studies where HPV16 E2 fused to GFP and CCHCR1 fused to mCherry were transiently expressed in HaCaT cells (Fig. 4). The fluorescence revealed that the expression pattern of CCHCR1 consists in punctuated signals throughout the cytoplasm. This distribution is coherent with observations made in other cell lines [21], [22] and with its endogenous expression pattern [23]. The E2 protein from HPV16 displays a diffuse expression pattern primarily concentrated in the nucleus and also present in the cytoplasm as previously described [24]. When both proteins were co-expressed, the distribution of HPV16 E2 was drastically modified whereas CCHCR1 was unaffected. Indeed, most of the E2 protein massively redistributed into the cytoplasm, where it co-localized with CCHCR1 in the dot-like structures typical of the CCHCR1 expression pattern. A decrease in E2 nuclear accumulation was concomitantly observed (Fig. 4). To verify that the redistribution of the HPV16 E2 protein resulted from its direct interaction with CCHCR1, we tested the HPV16 E2I73A mutant protein, defective for CCHCR1 binding. This mutant conserved its natural localization, predominantly nuclear, upon co-expression with CCHCR1 (Fig. 4). Similar experiments were conducted with a panel of non-interacting E2 proteins from different HPV genotypes, and no change in their subcellular distribution could be observed in the presence of CCHCR1 (Fig. 5). Altogether, these results show a strict correlation between the ability of CCHCR1 to induce E2 subcellular redistribution and its binding capacity to E2, thereby indicating that the redistribution of HPV16 E2 is indeed mediated by direct interaction with CCHCR1. The cytoplasmic docking of HPV16 E2 resulting from its association with CCHCR1 is likely to reinforce the inhibition of E2's nuclear functions.

**Figure 6:**
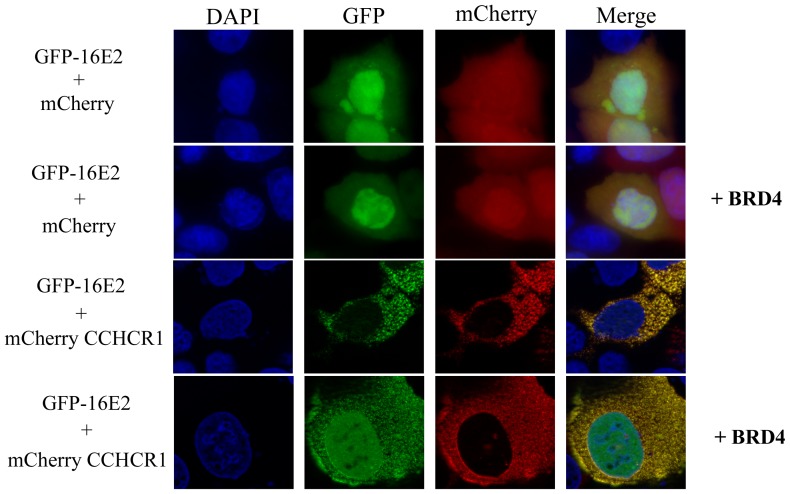
Intermediate pattern of HPV16 E2 distribution in the presence of CCHCR1 and BRD4. HaCaT cells were transfected with GFP-16E2 and mCherry-CCHCR1, untagged BRD4 or both together. After fixation, the cells were subjected to fluorescence microscopy after counterstaining of the nucleus with DAPI. HPV16 E2 subcellular localization is not affected by the expression of BRD4. In presence of both CCHCR1 and BRD4, HPV16 E2 distributed both in the nucleus and in CCHCR1-containing cytoplasmic dots.

As well, as discussed above, there is a competition between BRD4 and CCHCR1 for the interaction with HPV16 E2. In contrast to CCHCR1, BRD4 expression does not alter the nuclear localization of HPV16 E2, in line with an interaction occurring in the nucleus (Fig. 6). Upon co-expression with both BRD4 and CCHCR1 proteins, HPV16 E2 displayed an intermediate pattern combining a nuclear localization and a cytoplasmic distribution confined in CCHCR1-containing dots (Fig. 6). These results suggest that the competition between BRD4 and CCHCR1 for the binding to HPV16 E2 impacts on its subcellular localization. Overall, our results suggest that both the transcriptional functions and the subcellular distribution of HPV16 E2 might depend upon the proportion of BRD4 and CCHCR1 present in keratinocytes along the differentiating epithelium.

## Discussion

In the present study, we have identified the human protein CCHCR1 as a specific interactor of the E2 protein from HPV16, the prevailing genotype in HPV-associated cancers. CCHCR1 is unable to interact with any of the other mucosal HPV E2 proteins tested, suggesting that its interaction with the HPV16 E2 protein might contribute to some particular pathogenic traits of this HPV genotype in the mucosal context. Given the high prevalence of the HPV16 genotype in HPV-associated cancers, both in the genital sphere and in the oral area, one can hypothesize that such a specific interaction could potentially underlie its propensity to give rise to lesions evolving toward cancer. Indeed, the oncogenic power of HPV16 relies primarily on the E6 and E7 oncoproteins, whose immortalizing activities are basically shared by all mucosal high-risk HPV, and therefore do not allow rationalizing the over-representation of HPV16 in cancers. Other factors or mechanisms must play a role in HPV16-specific oncogenic trait, and the specific interaction between CCHCR1 and HPV16 E2 could be one such factor.

Current evidence indicates that CCHCR1 is associated with Psoriasis, a disease with hyperproliferative lesions of the skin. The observation that CCHCR1 specifically interacts with the E2 protein from HPV16, which is a mucosal HPV type, indicates that CCHCR1 probably has also a role in hyperproliferative lesions of keratinized epithelia other than skin, like the genital or oral epithelia.

We were surprised to find out that the binding interface of CCHCR1 on HPV16 E2 overlaps that of BRD4, which, unlike CCHCR1, binds to all the E2 proteins tested so far. Both interactions seem to span over the same surface of the N-terminal alpha-helices, which are greatly conserved among the E2 proteins. The specific aspect of the interaction between CCHCR1 and HPV16 E2 therefore constitutes a paradox. Searches for HPV16 E2-specific exposed amino acids failed to identify a key position for CCHCR1 binding. Another hypothesis is that this interaction requires the dimerization of E2 N-terminal domain, which could support the specificity toward HPV16. Indeed, in 2000 Anston *et al*. resolved the structure of the N-terminal domain of HPV16 E2, and showed that this domain by itself associates into dimers in solution, which they claim could be important for interactions with viral and cellular proteins [19]. They further showed that substitution of a number of amino acids leads to dimer disruption, among which amino acids R37 and I73, that we demonstrate here as being crucial for the interaction with CCHCR1. By contrast, during determination of the crystal structure the N-terminal domains of E2 from HPV11 and HPV18, it had been specified that each of these domains was fully monomeric, with no dimers formed in solution, while harboring a similar fold as HPV16 N-terminal domain [25], [26]. Therefore, only the N-terminal domain of HPV16 E2 seems to be able to dimerize, even though it requires conserved amino acids. It is thus conceivable that the binding specificity to CCHCR1 could be brought by a particular surface on the dimer of HPV16 E2 N-terminal domain, as well as by specific amino acids motifs scattered over each monomer.

The shared interaction surface of BRD4 and CCHCR1 leads to their competitive binding on HPV16 E2. BRD4 is essential for the transcriptional properties of E2, and disrupting this interaction is considered a promising anti-HPV strategy [3], [27]. We show here that the competition with CCHCR1 interferes with the positive role of BRD4 on E2 transcriptional properties. The transcriptional functions of E2 first operate at the level of viral genome, where E2 regulates the early and late promoters. Moreover, E2 was also shown to regulate cellular genes, mostly impacting the host differentiation program and thereby assisting implementation of the productive cycle (see a review in [4]). We demonstrate that HPV16 E2 drastically activates the expression of the early differentiation marker K10, which corroborates previous reports showing that the E2 protein from HPV16 stimulates epithelial differentiation in HaCaT [28]. The regulation of K10 by HPV16 E2 is likely to be transcriptional, albeit not direct since no E2 binding sites were identified in the K10 promoter. In line with this assumption, we observed that the I73A mutated HPV16 E2 protein failed to activate K10 transcription (data not shown). The activation of K10 by HPV16 is strongly inhibited in the presence of CCHCR1, which would be related to its competitive binding with BRD4 and subsequent reduction of E2 transcriptional activation potential.

Clues about the functional impact of the interaction between HPV16 E2 and CCHCR1 *in vivo* emerged from the study of keratinocytes differentiation. The keratinocyte growth and differentiation switch is tightly regulated by several mechanisms: as cells move through distinct epidermal layers, they are converted from proliferative, undifferentiated keratinocytes into highly differentiated non-dividing post-mitotic cells. In the context of the HPV life cycle, the virus requires that the host cells undertake differentiation for genome amplification and virion production. While the two HPV oncoproteins E6 and E7 induce continuous cell proliferation, E2 acts to counter-balance their effects, first by repressing their expression and, as described here, by promoting keratinocyte differentiation. In the case of HPV16, the influence of CCHCR1 could interfere with the induction of the differentiation program, leaving a window of opportunity for an exaggerated stimulation of proliferation by E6 and E7. It might therefore account for the enhanced tendency of HPV16-associated lesions to produce uncontrolled hyperplasia, with a high risk to progress toward cancers. CCHCR1 influence would thus favor the early steps of HPV16 carcinogenic conversion, preceding the potential disruption of E2 gene by integration of the viral genome into the host chromosome.

In line with this hypothesis, several links between CCHCR1 and HPV-associated cervical cancer were detected. Santin and colleagues identified CCHCR1 (herein named C6orf18) among many other genes overexpressed in primary cervical cancer cultures when compared to normal cervical keratinocytes [29]. More recently, an increase in CCHCR1 expression was observed in neoplastic cervical High-grade squamous intraepithelial lesions associated with HPV16 [30].

Furthermore, our study provides evidence that a strong delocalization of the HPV16 E2 protein in the cytoplasm results from its interaction with CCHCR1. Such cytoplasmic docking would reduce the amount of HPV16 E2 accessible to the nucleus and therefore interfere with its nuclear functions such as the regulation of viral and cellular (K10) genes expression, or the activation of viral DNA replication. It can as well impact the proper segregation of viral genomes during mitosis, which is dependent on E2 binding both the viral genome and BRD4. We also speculate that such a relocalization of HPV16 E2 in cytoplasmic dot-like structures could promote other functions of E2 operating in the cytoplasm. In line with this hypothesis, the comparative interactomic study of the E2 proteins recently uncovered the functional targeting by E2 of cell processes effective in the cytoplasm, such as vesicles trafficking between intracellular organelles [10]. This led to the proposal that the E2 proteins exert some activities in the cytoplasm, beyond their acknowledged nuclear functions. The interaction with CCHCR1 could well support such cytoplasmic activity of HPV16 E2, which would require further investigation.

It has to be stressed that, in contrast to the isoform collected from yeast two-hybrid, the full length CCHCR1 does not interact with HPV16 E2 (Fig. S5), advocating for an isoform-specific interaction. This is in line with previous studies demonstrating that several of the functions of CCHCR1 are isoform-specific [23]. The E2-interacting CCHCR1 isoform is only poorly expressed in proliferating HaCaT cells. We hypothesize that its level increases along the differentiating epithelium, where its interaction with HPV16 E2 would both affect HPV16 E2 subcellular distribution, and oppose E2 nuclear functions such as the stimulation of keratinocyte differentiation.

The functional repercussions of the specific interaction characterized in the present study are likely to impact on the pathogenesis of HPV16, and as such could be envisioned as a unique biomarker of HPV16 infection.

## Supporting Information

Figure S1 **Expression levels of Glc1 and Glc2 fusion proteins in GPCA conditions.** 293 T were transfected with the expression plasmids for Glc2 (A) and Glc1 (B) fusion proteins in the same conditions than the GPCA assay. Western blot analysis was performed on total cell lysate using anti-Gaussia polyclonal antiboby (Biolabs E8023S, 1/2500). Note that the anti-Gaussia luciferase antiboby detects the Gaussia C-terminal fragment (Glc2 fusion proteins) far less efficiently than the N-terminal Gaussia fragment (Glc1-fusion proteins), precluding any direct comparison between the expression levels of Glc1 and Glc2 proteins in GPCA conditions. The detection of Glc2-E2 fusion proteins is particularly difficult for the α-type HPV (left part of the upper panel) due to background bands migrating around the same sizes marked by an asterisk (*). NT stands for Not Transfected.
(TIF)

Figure S2 **Expression levels of Glc1 and Glc2 fusion proteins in GPCA conditions.** (A) 293 T were transfected with the expression plasmids for the Glc2 fusion proteins with the various mutated HPV16E2 in Figure 2 in the same condition as GPCA. Proteins were detected by western blot on total cell lysate using anti-Gaussia polyclonal antiboby (Biolabs E8023S, 1/2500). Note that an unspecific band (marked by an asterisk *) co-migrating with deleted forms of 16E2 proteins (16E2 ΔH1, ΔH1-2 and ΔH1-2-3) interfered with their detection. (B) 293 T were transfected with the expression plasmids for the Glc2 16 E2 or Glc2-11 E2 fusion proteins together with Glc1-BRD4, in the presence or not of 3XFLAG CCHCR1 in the same conditions as Figure 2-F (0,5 μg of each plasmid). Proteins were detected by western blot on total cell lysate using anti-Gaussia polyclonal antiboby (Biolabs E8023S, 1/2500) or Mouse anti-Flag (1∶5000, Sigma). NT stands for Not Transfected.
(TIF)

Figure S3 **CCHCR1 does not interfere with the activation of HPV18 E2-dependent transcription by BRD4.** HaCaT cells were transfected with an E2-reponsive luciferase reporter plasmid (pTK6E2BS) in the presence of HPV18 E2, BRD4 plus CCHCR1 where indicated. Fold activation are given relative to promoter activity without E2.
(TIF)

Figure S4 **The I73A mutated HPV16 E2 protein is unable to active K10 transcription.** HaCaT cells were transfected by 3XFLAG-HPV16 E2 I73A and CCHCR1 expression plasmids as indicated and the subsequent effect of the mRNA levels of K10 and K14 was monitored by qRT-PCR.
(TIF)

Figure S5 **Isoform-specific binding of CCHCR1 to 16E2.** A. Interaction between 16E2 and the different isoforms of CCHCR1 was assessed by GPCA as in figure 1. B.293 T cells were cotransfected with expression plasmids for flag-tagged full-length or shorter “E2-interacting” CCHCR1 isoform, and GFP-HPV16 E2. Cells were lysed and subjected to immunoprecipitation (IP) using anti Flag antibody followed by western Blotting (WB) with anti FLAG or anti GFP antibodies.
(TIF)

## References

[1] zur Hausen H (2009) Papillomaviruses in the causation of human cancers - a brief historical account. Virology 384: 260–265. 19135222 10.1016/j.virol.2008.11.046

[2] Doorbar J (2006) Molecular biology of human papillomavirus infection and cervical cancer. Clin Sci (Lond) 110: 525–541. 16597322 10.1042/CS20050369

[3] D'Abramo CM, Archambault J (2011) Small molecule inhibitors of human papillomavirus protein - protein interactions. Open Virol J 5: 80–95. 21769307 10.2174/1874357901105010080 PMC3137155

[4] Muller M, Demeret C (2012) The HPV E2-Host Protein-Protein Interactions: A Complex Hijacking of the Cellular Network. Open Virol J 6: 173–189. 23341853 10.2174/1874357901206010173 PMC3547520

[5] Asumalahti K, Laitinen T, Itkonen-Vatjus R, Lokki ML, Suomela S, et al (2000) A candidate gene for psoriasis near HLA-C, HCR (Pg8), is highly polymorphic with a disease-associated susceptibility allele. Hum Mol Genet 9: 1533–1542. 10888604 10.1093/hmg/9.10.1533

[6] Asumalahti K, Veal C, Laitinen T, Suomela S, Allen M, et al (2002) Coding haplotype analysis supports HCR as the putative susceptibility gene for psoriasis at the MHC PSORS1 locus. Hum Mol Genet 11: 589–597. 11875053 10.1093/hmg/11.5.589

[7] Tiala I, Wakkinen J, Suomela S, Puolakkainen P, Tammi R, et al (2008) The PSORS1 locus gene CCHCR1 affects keratinocyte proliferation in transgenic mice. Hum Mol Genet 17: 1043–1051. 18174193 10.1093/hmg/ddm377

[8] Suomela S, Elomaa O, Skoog T, Ala-aho R, Jeskanen L, et al (2009) CCHCR1 is up-regulated in skin cancer and associated with EGFR expression. PLoS One 4: e6030. 19551138 10.1371/journal.pone.0006030 PMC2696036

[9] Wu SY, Chiang CM (2007) The double bromodomain-containing chromatin adaptor Brd4 and transcriptional regulation. J Biol Chem 282: 13141–13145. 17329240 10.1074/jbc.R700001200

[10] Muller M, Jacob Y, Jones L, Weiss A, Brino L, et al (2012) Large scale genotype comparison of human papillomavirus e2-host interaction networks provides new insights for e2 molecular functions. PLoS Pathog 8: e1002761. 22761572 10.1371/journal.ppat.1002761 PMC3386243

[11] Bellanger S, Demeret C, Goyat S, Thierry F (2001) Stability of the human papillomavirus type 18 E2 protein is regulated by a proteasome degradation pathway through its amino-terminal transactivation domain. J Virol 75: 7244–7251. 11461997 10.1128/JVI.75.16.7244-7251.2001 PMC114960

[12] Boukamp P, Petrussevska RT, Breitkreutz D, Hornung J, Markham A, et al (1988) Normal keratinization in a spontaneously immortalized aneuploid human keratinocyte cell line. J Cell Biol 106: 761–771. 2450098 10.1083/jcb.106.3.761 PMC2115116

[13] Micallef L, Belaubre F, Pinon A, Jayat-Vignoles C, Delage C, et al (2009) Effects of extracellular calcium on the growth-differentiation switch in immortalized keratinocyte HaCaT cells compared with normal human keratinocytes. Exp Dermatol 18: 143–151. 18637039 10.1111/j.1600-0625.2008.00775.x

[14] Livak KJ, Schmittgen TD (2001) Analysis of relative gene expression data using real-time quantitative PCR and the 2(-Delta Delta C(T)) Method. Methods 25: 402–408. 11846609 10.1006/meth.2001.1262

[15] Olejnik-Schmidt AK, Schmidt MT, Kedzia W, Gozdzicka-Jozefiak A (2008) Search for cellular partners of human papillomavirus type 16 E2 protein. Arch Virol 153: 983–990. 18305892 10.1007/s00705-008-0061-6

[16] Cassonnet P, Rolloy C, Neveu G, Vidalain PO, Chantier T, et al (2011) Benchmarking a luciferase complementation assay for detecting protein complexes. Nat Methods 8: 990–992. 22127214 10.1038/nmeth.1773

[17] Venkatesan K, Rual JF, Vazquez A, Stelzl U, Lemmens I, et al (2009) An empirical framework for binary interactome mapping. Nat Methods 6: 83–90. 19060904 10.1038/nmeth.1280 PMC2872561

[18] Abbate EA, Voitenleitner C, Botchan MR (2006) Structure of the papillomavirus DNA-tethering complex E2:Brd4 and a peptide that ablates HPV chromosomal association. Mol Cell 24: 877–889. 17189190 10.1016/j.molcel.2006.11.002

[19] Antson AA, Burns JE, Moroz OV, Scott DJ, Sanders CM, et al (2000) Structure of the intact transactivation domain of the human papillomavirus E2 protein. Nature 403: 805–809. 10693813 10.1038/35001638

[20] Schweiger MR, You J, Howley PM (2006) Bromodomain protein 4 mediates the papillomavirus E2 transcriptional activation function. J Virol 80: 4276–4285. 16611886 10.1128/JVI.80.9.4276-4285.2006 PMC1472042

[21] Corbi N, Bruno T, De Angelis R, Di Padova M, Libri V, et al (2005) RNA polymerase II subunit 3 is retained in the cytoplasm by its interaction with HCR, the psoriasis vulgaris candidate gene product. J Cell Sci 118: 4253–4260. 16141233 10.1242/jcs.02545

[22] Tiala I, Suomela S, Huuhtanen J, Wakkinen J, Holtta-Vuori M, et al (2007) The CCHCR1 (HCR) gene is relevant for skin steroidogenesis and downregulated in cultured psoriatic keratinocytes. J Mol Med (Berl) 85: 589–601. 17221218 10.1007/s00109-006-0155-0

[23] Tervaniemi MH, Siitonen HA, Soderhall C, Minhas G, Vuola J, et al (2012) Centrosomal localization of the psoriasis candidate gene product, CCHCR1, supports a role in cytoskeletal organization. PLoS One 7: e49920. 23189171 10.1371/journal.pone.0049920 PMC3506594

[24] Blachon S, Bellanger S, Demeret C, Thierry F (2005) Nucleo-cytoplasmic shuttling of high risk human Papillomavirus E2 proteins induces apoptosis. J Biol Chem 280: 36088–36098. 16135518 10.1074/jbc.M505138200

[25] Abbate EA, Berger JM, Botchan MR (2004) The X-ray structure of the papillomavirus helicase in complex with its molecular matchmaker E2. Genes Dev 18: 1981–1996. 15289463 10.1101/gad.1220104 PMC514179

[26] Wang Y, Coulombe R, Cameron DR, Thauvette L, Massariol MJ, et al (2004) Crystal structure of the E2 transactivation domain of human papillomavirus type 11 bound to a protein interaction inhibitor. J Biol Chem 279: 6976–6985. 14634007 10.1074/jbc.M311376200

[27] Helfer CM, Wang R, You J (2013) Analysis of the Papillomavirus E2 and Bromodomain Protein Brd4 Interaction Using Bimolecular Fluorescence Complementation. PLoS One 8: e77994. 24205059 10.1371/journal.pone.0077994 PMC3808292

[28] Burns JE, Walker HF, Schmitz C, Maitland NJ (2010) Phenotypic effects of HPV-16 E2 protein expression in human keratinocytes. Virology 401: 314–321. 20347469 10.1016/j.virol.2010.03.002

[29] Santin AD, Zhan F, Bignotti E, Siegel ER, Cane S, et al (2005) Gene expression profiles of primary HPV16- and HPV18-infected early stage cervical cancers and normal cervical epithelium: identification of novel candidate molecular markers for cervical cancer diagnosis and therapy. Virology 331: 269–291. 15629771 10.1016/j.virol.2004.09.045

[30] Pacholska-Bogalska J, Myga-Nowak M, Ciepluch K, Jozefiak A, Kwasniewska A, et al (2012) Analysis of the coding sequence and expression of the coiled-coil alpha-helical rod protein 1 gene in normal and neoplastic epithelial cervical cells. Int J Mol Med 29: 669–676. 22218424 10.3892/ijmm.2012.877 PMC3577136

