# Supplementary figures and images for: CCHCR1 Interacts Specifically with the E2 Protein of Human Papillomavirus Type 16 on a Surface Overlapping BRD4 Binding
Source: PLoS One. 2014 Mar 24;9(3):e92581. doi: 10.1371/journal.pone.0092581 (PMC3963918; doi:10.1371/journal.pone.0092581)

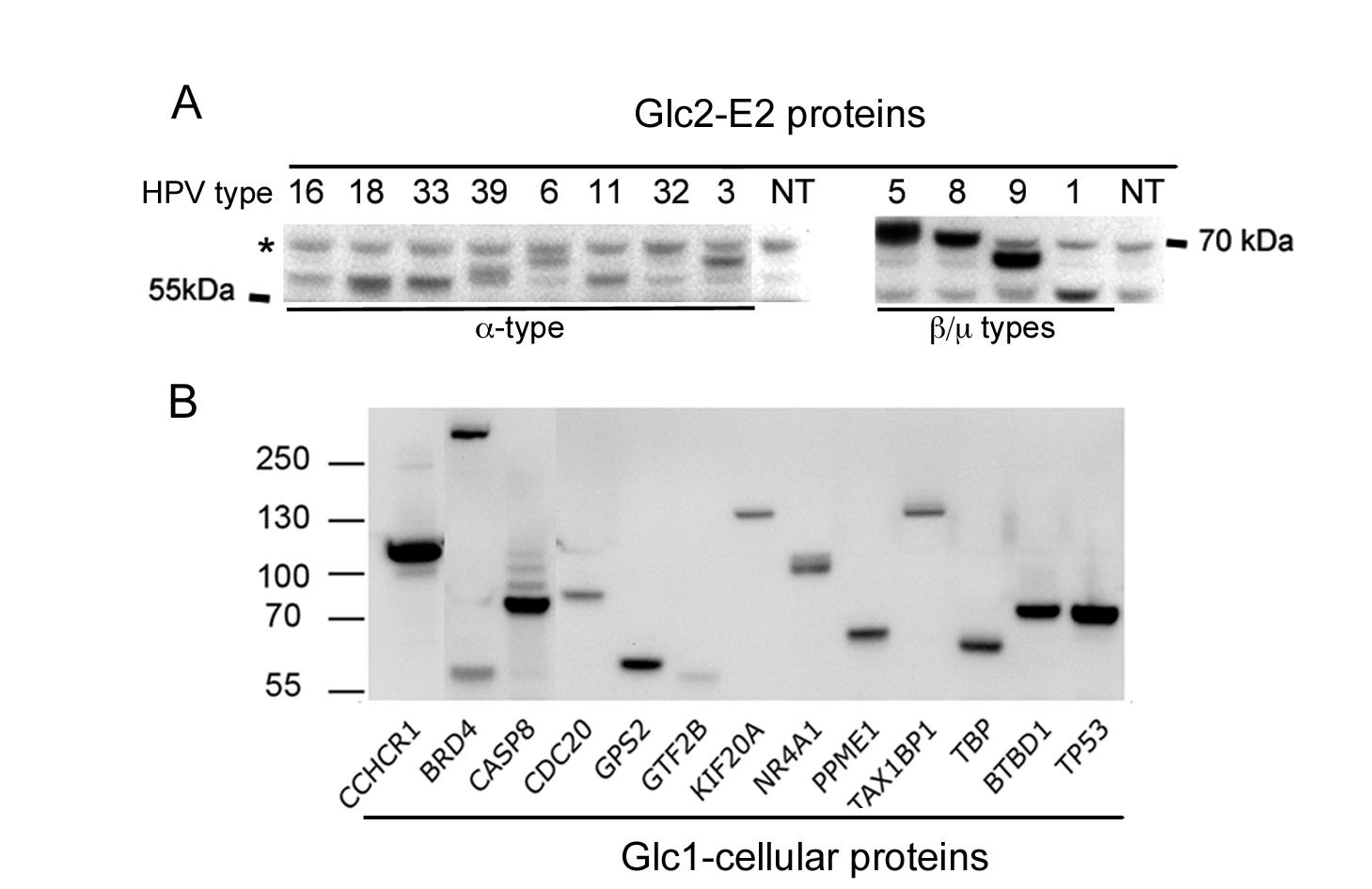

Supplement: Figure S1 — Expression levels of Glc1 and Glc2 fusion proteins in GPCA conditions. 293 T were transfected with the expression plasmids for Glc2 (A) and Glc1 (B) fusion proteins in the same conditions than the GPCA assay. Western blot analysis was performed on total cell lysate using anti-Gaussia polyclonal antiboby (Biolabs E8023S, 1/2500). Note that the anti-Gaussia luciferase antiboby detects the Gaussia C-terminal fragment (Glc2 fusion proteins) far less efficiently than the N-terminal Gaussia fragment (Glc1-fusion proteins), precluding any direct comparison between the expression levels of Glc1 and Glc2 proteins in GPCA conditions. The detection of Glc2-E2 fusion proteins is particularly difficult for the α-type HPV (left part of the upper panel) due to background bands migrating around the same sizes marked by an asterisk (*). NT stands for Not Transfected. (TIF) [file pone.0092581.s001.tif]

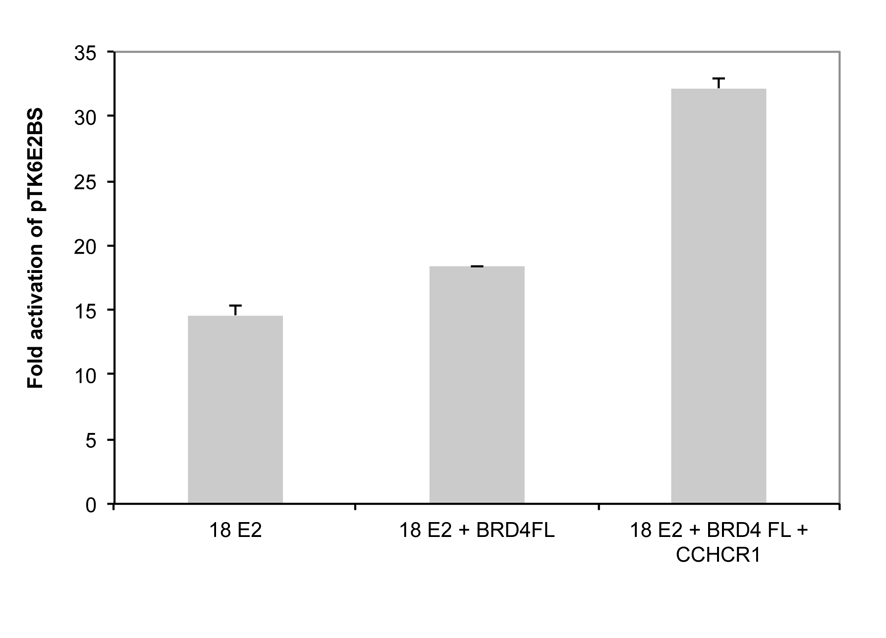

Supplement: Figure S3 — CCHCR1 does not interfere with the activation of HPV18 E2-dependent transcription by BRD4. HaCaT cells were transfected with an E2-reponsive luciferase reporter plasmid (pTK6E2BS) in the presence of HPV18 E2, BRD4 plus CCHCR1 where indicated. Fold activation are given relative to promoter activity without E2. (TIF) [file pone.0092581.s003.tif]

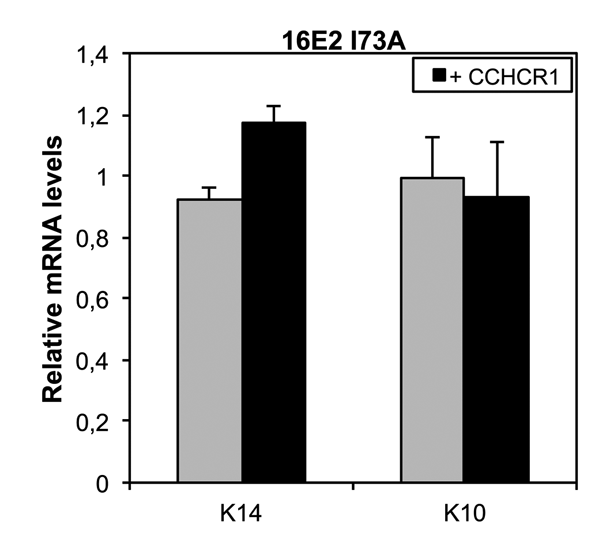

Supplement: Figure S4 — The I73A mutated HPV16 E2 protein is unable to active K10 transcription. HaCaT cells were transfected by 3XFLAG-HPV16 E2 I73A and CCHCR1 expression plasmids as indicated and the subsequent effect of the mRNA levels of K10 and K14 was monitored by qRT-PCR. (TIF) [file pone.0092581.s004.tif]

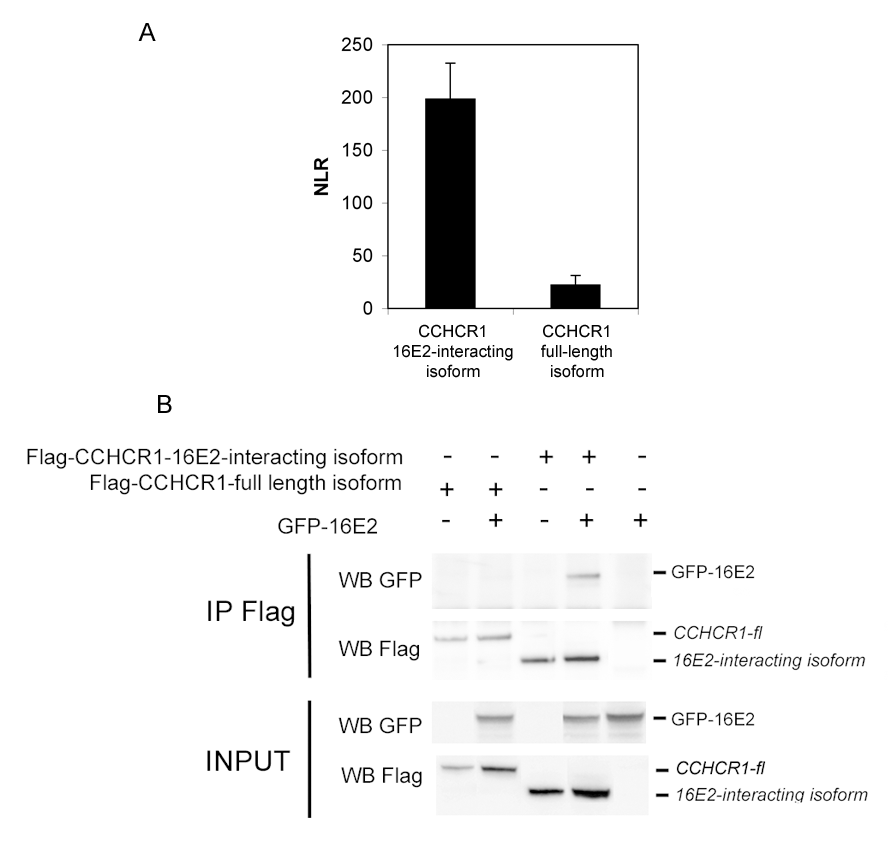

Supplement: Figure S5 — Isoform-specific binding of CCHCR1 to 16E2. A. Interaction between 16E2 and the different isoforms of CCHCR1 was assessed by GPCA as in figure 1. B.293 T cells were cotransfected with expression plasmids for flag-tagged full-length or shorter “E2-interacting” CCHCR1 isoform, and GFP-HPV16 E2. Cells were lysed and subjected to immunoprecipitation (IP) using anti Flag antibody followed by western Blotting (WB) with anti FLAG or anti GFP antibodies. (TIF) [file pone.0092581.s005.tif]
